# Direct Evidence of Abortive Lytic Infection-Mediated Establishment of Epstein-Barr Virus Latency During B-Cell Infection

**DOI:** 10.3389/fmicb.2020.575255

**Published:** 2021-01-21

**Authors:** Tomoki Inagaki, Yoshitaka Sato, Jumpei Ito, Mitsuaki Takaki, Yusuke Okuno, Masahiro Yaguchi, H. M. Abdullah Al Masud, Takahiro Watanabe, Kei Sato, Shingo Iwami, Takayuki Murata, Hiroshi Kimura

**Affiliations:** ^1^Department of Virology, Nagoya University Graduate School of Medicine, Nagoya, Japan; ^2^Precursory Research for Embryonic Science and Technology (PRESTO), Japan Science and Technology Agency, Kawaguchi, Japan; ^3^Division of Systems Virology, Department of Infectious Disease Control, International Research Center for Infectious Diseases, Institute of Medical Science, The University of Tokyo, Tokyo, Japan; ^4^Mathematical Biology Laboratory, Department of Biology, Faculty of Sciences, Kyushu University, Fukuoka, Japan; ^5^Medical Genomics Center, Nagoya University Hospital, Nagoya, Japan; ^6^Department of Microbiology, Faculty of Biological Sciences, University of Chittagong, Chattogram, Bangladesh; ^7^Core Research for Evolutional Science and Technology (CREST), Japan Science and Technology Agency, Kawaguchi, Japan; ^8^MIRAI, Japan Science and Technology Agency, Kawaguchi, Japan; ^9^Department of Virology and Parasitology, Fujita Health University School of Medicine, Toyoake, Japan

**Keywords:** EBV, pre-latent phase, abortive lytic infection, fate mapping, neo virology

## Abstract

Viral infection induces dynamic changes in transcriptional profiles. Virus-induced and antiviral responses are intertwined during the infection. Epstein-Barr virus (EBV) is a human gammaherpesvirus that provides a model of herpesvirus latency. To measure the transcriptome changes during the establishment of EBV latency, we infected EBV-negative Akata cells with EBV-EGFP and performed transcriptome sequencing (RNA-seq) at 0, 2, 4, 7, 10, and 14 days after infection. We found transient downregulation of mitotic division-related genes, reflecting reprogramming of cell growth by EBV, and a burst of viral lytic gene expression in the early phase of infection. Experimental and mathematical investigations demonstrate that infectious virions were not produced in the pre-latent phase, suggesting the presence of an abortive lytic infection. Fate mapping using recombinant EBV provided direct evidence that the abortive lytic infection in the pre-latent phase converges to latent infection during EBV infection of B-cells, shedding light on novel roles of viral lytic gene(s) in establishing latency. Furthermore, we find that the BZLF1 protein, which is a key regulator of reactivation, was dispensable for abortive lytic infection in the pre-latent phase, suggesting the divergent regulation of viral gene expressions from a productive lytic infection.

## Introduction

Numerous signaling events are triggered during the first few days of viral infection. Virus entry into the target cells results in the activation of cellular signaling pathways (Hiscott et al., 2001). Concomitantly, viral pathogens are recognized by host sensor molecules, leading to activation of immune responses (Malmgaard, 2004; Perry et al., 2005). Viruses rewire and modulate this interspecies interaction to meet their own needs and, consequently, establish latency in their host cells.

Epstein-Barr virus (EBV), a gammaherpesvirus, is a widely dispersed enveloped virus that infects > 90% of adults worldwide. It is associated with several types of human malignancies with an incidence of 200,000 EBV-related cancers estimated annually (Cohen et al., 2011). Although most primary EBV infections are asymptomatic, EBV infection can cause infectious mononucleosis, especially when primary infection is delayed until late adolescence or early adulthood (Cohen, 2000). EBV establishes latent infection primarily in B-cells and typically persists for the life of the individual (Babcock et al., 1998; Young and Rickinson, 2004) although the virus can demonstrate both latent and lytic cycles in lymphocytes after primary infection. Thus, EBV provides a model system for studying how viruses, and particularly herpesviruses, establish latency in the cells.

In the latent state, the EBV genome is maintained as circular plasmids forming nucleosomal structures with histones, which express a limited number of viral gene products (Adams, 1987). Therefore, no production of virus particles occurs during latent infection. Periodically, latent EBV switches from the latent stage into the lytic cycle to produce progeny viruses within its host cell. During the lytic infection, all EBV genes are expressed in a strictly regulated temporal cascade, and the circular genomes are amplified by the viral replication machinery, generating infectious virions (Tsurumi et al., 2005; Sato and Tsurumi, 2010).

Accumulating evidence shows the abortive lytic cycle as a third state of EBV infection occurring in the pre-latent phase of EBV primary infection (Wen et al., 2007; Kalla et al., 2010; Jochum et al., 2012a,b; Sato et al., 2017; Wang et al., 2019) and within EBV-associated tumors (Hong et al., 2005, 2009; Murata et al., 2019; Okuno et al., 2019). In this state, the full lytic program is not induced due to an incomplete expression cascade of viral lytic genes; thus, infectious particles are not produced. Abortive lytic replication and its associated viral gene expressions are implicated in the pathogenesis of EBV-associated malignancies (Ma et al., 2011; Munz, 2019; Okuno et al., 2019). Transient lytic gene expression in newly infected B-cells is essential for the emergence of lymphoblastoid cell lines (Altmann and Hammerschmidt, 2005).

mRNA expression profiling by RNA sequencing (RNA-seq) and quantitative PCR (qPCR) analysis has provided important information on viral gene expression throughout these states of EBV-infected cells. However, such “snapshot” data lack temporal resolution, rendering them unsuitable to address the fate of infected cells during EBV infection. Continuous analysis is crucial to decoding this dynamic and heterogenous process. In this study, we address the fate of infected cells, which exhibit an abortive lytic infection, during EBV infection and record lytic gene expression by fate mapping with recombinant EBV. Our findings reveal that EBV is able to establish latency in B-cells via abortive lytic infection in the pre-latent phase.

## Results

### Expression Profiles of EBV-Infected Cells During the Pre-latent Phase

**FIGURE 1:**
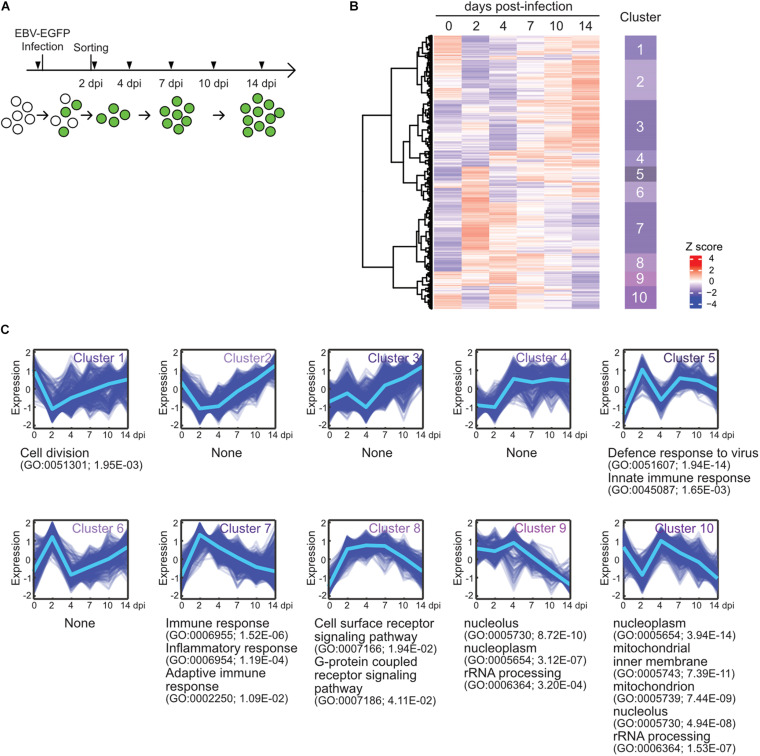
EBV infection caused reprogramming of cell growth. **(A)** Workflow of sample collection for RNA-seq analysis. Akata(–) cells were infected with EBV-EGFP. The infected cells were collected by FACS sorting at 2 dpi, and portions of infected cells were harvested at the indicated time points (arrowheads). **(B)** Heat map showing gene expression changes during EBV infection of Akata(–) cells. The 5,000 most diversely expressed genes of both the host and virus are included. Color indicates the normalized expression level (*Z* score). Gene clusters are indicated on the right side of the heat map. **(C)** Temporal changes in gene expression for each cluster. The changes of respective genes (blue) and the mean value (light blue) are plotted. GO enrichment analysis was performed in each cluster, and the representative results (GO terms, GO Ids, and adjusted *p*-values) are shown on the right side of the graph.

To elucidate the gene expression dynamics during the course of EBV infection, we performed a time-course transcriptome analysis on EBV-infected cells (Figure 1A). Here, we used Akata cells, the Burkitt lymphoma cell line, instead of primary B-cells, because we focus on the infection-mediated transcriptional changes during EBV infection without EBV-driven transformation and further subsequent analysis using genetics. EBV-negative Akata(−) cells were infected with EBV-enhanced green fluorescent protein (EGFP), and infected cells were collected by fluorescence-activated cell sorting (FACS) at 2 days post-infection (dpi) by EGFP-positivity. Subsequently, transcriptome information of the infected cells was obtained at 2, 4, 7, 10, and 14 dpi by RNA-seq. Clustering analysis grouped genes into 10 clusters (Figure 1B), according to the temporal expression patterns across time points (Figure 1C). Gene ontology (GO) enrichment analysis was performed to interpret respective gene clusters (Figure 1C).

Cluster 1 displayed an immediate decrease in gene expression, followed by recovery. Likewise, cluster 10 expression levels immediately decreased and then rapidly increased. These clusters preferentially included genes involving cell division and energy- or bio-syntheses (i.e., mitochondrion and rRNA processing-related genes), suggesting that cell growth was suppressed after viral infection. This observation is consistent with previous findings that the rapid growth of EBV-infected cells is coupled with an increase in biomass for energy and the production of biosynthetic intermediates (McFadden et al., 2016). The Hammerschmidt laboratory also showed that infected cells did not divide within the first 3 days of infection but rapidly recommenced growth at 4 dpi, during EBV infection to naïve human B-cells (Pich et al., 2019). Clusters 9 and 10 displayed gradual decreases in their gene expressions after 4 dpi, corresponding to virus-mediated modulations of cellular processes, such as DNA replication, histone modification, and transcription. Thus, EBV reprogrammed the transcriptome of infected cells during the initial stage of infection even without immortalization, similar to EBV infection of naïve or resting B-cells (Mrozek-Gorska et al., 2019; Pich et al., 2019; Wang et al., 2019).

In agreement with data from EBV infection of human primary resting B-cells (Wang et al., 2019), clusters 5 and 7 displayed peak expression at 2 dpi enriched with genes involved in immune responses.

Cluster 8 included genes involved in the signaling pathways of cell surface receptors and G-protein coupled receptors, and its pattern was similar to viral gene expression (described below). Furthermore, DAVID Bioinformatics Resources (Huang et al., 2009) identified that protein tyrosine phosphatase non-receptor type 11 (PTPN11) interactors were enriched among upregulated proteins in EBV-infected Akata cells (Table 1). PTPN11 encodes the tyrosine phosphatase SHP2, which acts downstream of receptor tyrosine kinases, such as EGFR and FGFR, to affect survival and proliferation through the activation of the RAS/MAPK cascade (Chan and Feng, 2007). Indeed, Tang et al. (2019) report that PTPN11 is upregulated in EBV-transformed lymphoblasts. Thus, PTPN11 may play a critical role in latency establishment in the pre-latent phase of EBV infection although a molecular mechanism of this process remains obscure.

**TABLE 1:** BioGRID interactome analysis of top 300 upregulated genes at 2 dpi.

Term	Gene name	Count	*p*-value	FDR^*a*^
PTPN11	STAT5A, CEACAM1, FCGR2A, CYP1A1, PECAM1, GAB2, PILRA, LGALS9, FCGR2B, BCAR1	10	7.90 × 10^–^^6^	0.042
PIK3R1	CSF1R, FCGR2A, SRC, SH2D2A, DAB2IP, PECAM1, PTPN6, GAB2, BCAR1, TGFBR2	10	7.77 × 10^–^^5^	0.21
TRAF2	TNFRSF12A, MVP, RNASET2, CASP1, TNFSF10, TNFAIP3, TNFRSF14, TRAF1, CFLAR, TNFRSF1B, TNF	11	2.31 × 10^–^^4^	0.41
ISG15	UBA7, ELF3, DDX58, IFIT1, USP18	5	4.46 × 10^–^^4^	0.60
IFIT3	DDX58, ISG15, IFIT1, IFIT3, IFIT2	5	6.12 × 10^–^^4^	0.65

*^*a*^FDR, false discovery rate.*

### Transient Burst of Viral Lytic Gene Expression During the Pre-latent Phase of EBV Infection

**FIGURE 2:**
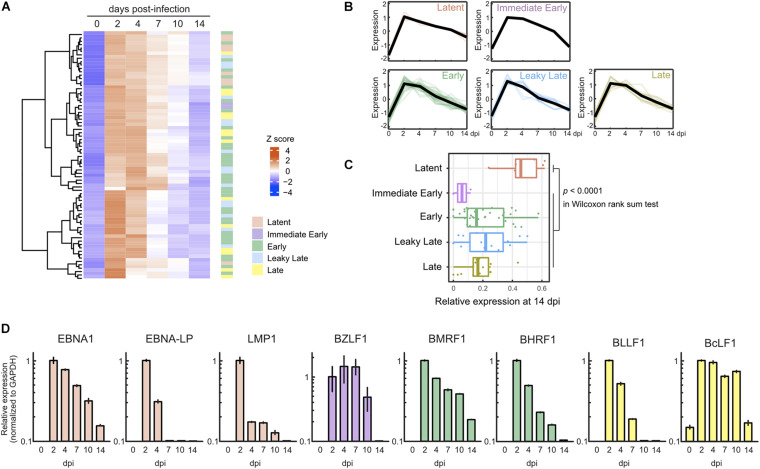
Transient burst of viral lytic gene expression in the pre-latent phase of EBV infection of Akata(–) cells. **(A)** Heat map showing the viral gene expression changes during EBV infection of Akata(–) cells. **(B)** Temporal change of viral gene expression in each kinetic lytic gene. The changes of respective genes and the mean values (black) are plotted. Viral gene expression kinetics are categorized into five groups: latent, immediate early, early, leaky late, and late (Djavadian et al., 2018). **(C)** Relative viral gene expression of latent, immediate early, early, leaky late, and late kinetics at 14 dpi. **(D)** Validation of viral gene expression by qRT-PCR. Viral gene expression was normalized to GAPDH expression. Results are presented as means ± SD of three independent experiments and are shown relative to gene expression at 2 dpi.

In parallel with cellular gene expression, viral genes are expressed in a dynamic but regulated manner during *de novo* infection of B-cells. Two days after infection, we detected almost all viral genes, including lytic genes, and most of these genes were suppressed by 7 dpi (Figures 2A,B). Consequently, the pattern of viral gene expression demonstrated latent infection at 14 dpi (Figure 2C). The expression of representative lytic genes was validated by qRT-PCR, and their transient burst also was confirmed (Figure 2D).

**FIGURE 3:**
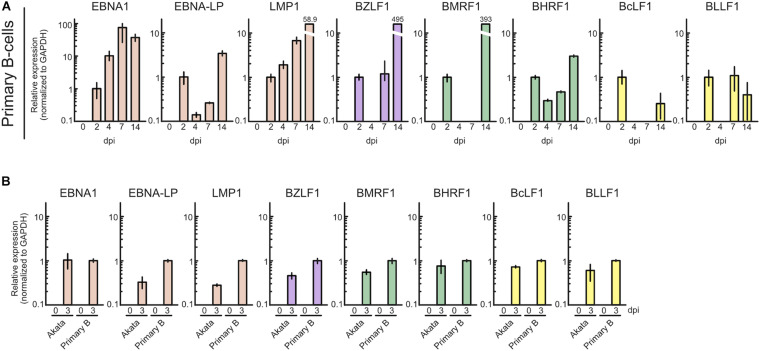
Profile of EBV gene expression in the pre-latent phase of EBV infection of primary B-cells. **(A)** Purified primary B-cells were infected with EBV-EGFP and harvested at 0, 2, 4, 7, or 14 dpi. EBV gene expression was assessed by qRT-PCR and normalized to GAPDH expression. Results are presented as means ± SD of three independent experiments and are shown relative to gene expression at 2 dpi. **(B)** Akata(–) or purified primary B-cells were infected with EBV-EGFP and harvested at 0 and 3 dpi. EBV gene expression was assessed by qRT-PCR and normalized to GAPDH expression. Results are presented as means ± SD of three independent experiments. Abbreviations: dpi, day post-infection.

To ascertain whether this phenomenon is specific to Akata cells, we also assessed lytic gene expression in primary B-cells with infected with EBV. Primary B-cells were isolated and infected with EBV-EGFP, and viral gene expression was assessed by qRT-PCR. As shown in Figure 3A, EBV lytic gene expression was detected in the pre-latent phase of EBV-infected primary B-cells, consistent with a previous report (Wang et al., 2019). We compared the expression level of EBV genes between Akata and primary B-cells (Figure 3B). The different pattern of lytic gene expression between Akata cells and primary B-cells may be due to the duration of latency establishment, properties of cells, or procedure of EBV infection. From these data, we conclude that abortive lytic EBV gene expression occurs during EBV infection.

### Infectious Virion Production Is Halted During the Pre-latent Phase

**FIGURE 4:**
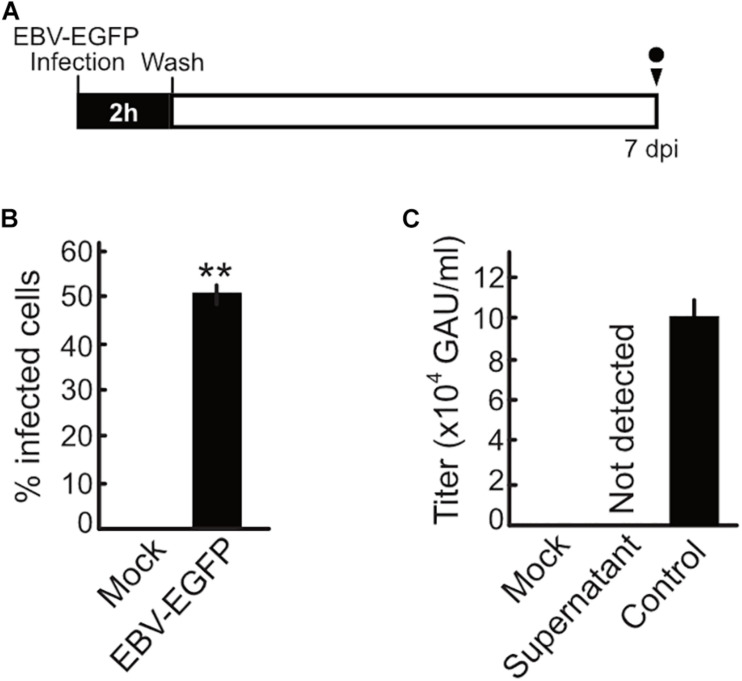
No progeny production was detected during the pre-latent phase of EBV infection. **(A)** Schematic schedule of this experiment. Akata(–) cells (1 × 10^6^ cells) were incubated with 1 × 10^6^ GAU of EBV-EGFP at room temperature for 2 h with agitation. Cells were extensively washed with PBS to remove unbound virus and then suspended in fresh medium. Cells and culture supernatant were harvested at 7 dpi. GAU, green Akata units. **(B)** Infected cells were quantified by FACS. Results shown are the means ± SD of three independent experiments. Double asterisks (**) indicate *p* < 0.01. **(C)** Virus titer in the supernatant was determined as described previously (Sato et al., 2019). Results shown are the means ± SD of three independent experiments. EBV-EGFP (1 × 10^5^ GAU/ml) was used as a positive control.

Upon EBV *de novo* infection, synthesis of the progeny virus was not observed in a previous study (Kalla et al., 2010). Our present study also confirmed this phenomenon (Figure 4). Akata(−) cells were infected with EBV and then washed with PBS after 2 h to remove unbound EBV inoculum. The cells were maintained and monitored for 7 dpi. Although infected cells were observed at 7 dpi, infectious virions were not detected in the supernatant at this time (Figure 4). We also confirm that the viral DNA genome was not detected in the supernatant harvested at 7 dpi (data not shown).

### Experimental Mathematical Investigation Reveals No Progeny Production During the Pre-latent Phase of EBV Infection

Next, we carried out an *in silico* simulation based on a mathematical model and estimated parameters from the experiments. Several studies on other viruses have established mathematical models that describe cell-free infection (Iwami et al., 2012, 2015). We propose the following mathematical models (Eqs. 1–4) considering the two opposite assumptions of the presence or absence of progeny virus production:

(1) $$\frac{d\,T\,\left(t\right)}{d\,t}=g\,T\,\left(t\right)\,\left(1-\frac{T\,\left(t\right)+I_{a\,r}\,\left(t\right)+I_{p\,r}\,\left(t\right)}{K}\right)-\beta \,T\,\left(t\right)\,V\,\left(t\right)$$

(2) $$\frac{d\,I_{a\,r}\,\left(t\right)}{d\,t}=\beta \,T\,\left(t\right)\,V\,\left(t\right)-\delta \,I_{a\,r}\,\left(t\right)$$

(3) $$\frac{d\,I_{p\,r}\,\left(t\right)}{d\,t}=\delta \,I_{a\,r}\,\left(t\right)+g\,I_{p\,r}\,\left(t\right)\,\left(1-\frac{T\,\left(t\right)+I_{a\,r}\,\left(t\right)+I_{p\,r}\,\left(t\right)}{K}\right)$$

(4) $$\frac{d\,V\,\left(t\right)}{d\,t}=p\,\left(I_{a\,r}\,\left(t\right)+I_{p\,r}\,\left(t\right)\right)-c\,V\,\left(t\right)$$

**FIGURE 5:**
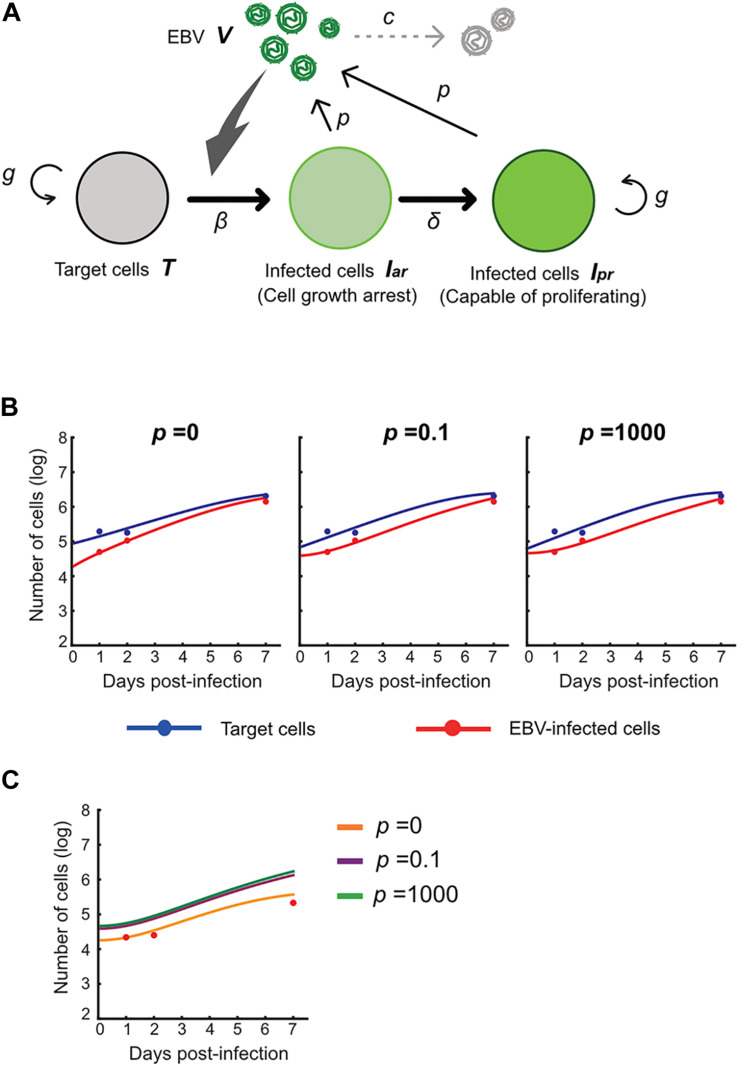
Experimental mathematical investigation of progeny production during the pre-latent phase of EBV infection. **(A)** A mathematical model for EBV infection of B cells is described. The parameter *g* is the growth rate of cells, *K* is the carrying capacity, β is the cell-free infection rate, δ is the reciprocal number of the cell growth arrest period, *p* is the progeny virus production rate, and *c* is the decay rate of EBV. Note that *p* = 0 corresponds with no progeny virus production. **(B)** With fixed values of *p* = 0, 0.1 or 1,000, we fit Eqs (3–6) to the experimental data and describe the solid curves of the best-fit solution. **(C)** The number of infected cells was calculated from the mathematical model with estimated parameters. Except in the case of *p* = 0, prediction by the mathematical model could not property reproduce actual EBV infection dynamics properly.

Here, *T*(*t*) is the number of Akata cells, and the parameters *g* and *K* are the growth rate and the carrying capacity of the cell culture, respectively. The variable *I*_*ar*_(*t*) is the number of EBV-infected cells with cell growth arrest, *I*_*pr*_(*t*) is that of all other EBV-infected cells, *V*(*t*) is the number of virions, β is the infection rate, δ is the reciprocal number of the cell growth arrest period (i.e., δ = 0.5 here), *p* is the progeny virus production rate, and *c* represents the viral decay rate. Initial values of the number of target and infected cells are given by *T*_0_ and *I*_*ar*,0_, respectively (see Figure 5A). In our data fitting, we estimate β, *T*_*0*_, and *I*_*ar;0*_ for *p* = 0, 0.1, and 1000, fixing the independently estimated values of *g*, *K*, and c as described in section “Growth Kinetics of Akata Cells and Viral Decay Kinetics” in “Materials and Methods” (see Figure 5B). Note that, for these algorithms, when progeny virus *p* equals 0, no virus progeny is produced. All estimated parameters are summarized in Table 2.

**TABLE 2:** Estimated parameters by fitting the mathematical model to experimental data.

Symbol	Unit	Value		
		
		*p* = 0	*p* = 0.1	*p* = 1,000
*g*	Day^–^^1^	7.33 × 10^–^^1^	7.33 × 10^–^^1^	7.33 × 10^–^^1^
*K*	Cell	6.51 × 10^6^	6.51 × 10^6^	6.51 × 10^6^
*c*	Day^–^^1^	2.44 × 10^–^^1^	2.44 × 10^–^^1^	2.44 × 10^–^^1^
β	(Day × cell)^–^^1^	2.46 × 10^–^^6^	8.53 × 10^4^	1.80 × 10^4^
*T*_0_	Cell	6.36 × 10^–^^7^	6.76 × 10^4^	3.88 × 10^4^
*I*_*ar*,0_	Cell	8.08 × 10^–^^11^	6.17 × 10^4^	4.62 × 10^4^

Using these estimated parameters, we further calculated EBV infection dynamics *in silico* under the assumption of removing the unbound virus and then compared our mathematical prediction and our experimental data under parallel conditions. Interestingly, as shown in Figure 5C, our model accurately described the actual EBV infection dynamics best in the case of *p* = 0, suggesting no progeny production in the pre-latent phase. Taken together, the transient expression of lytic genes during the pre-latent phase reflects an abortive lytic infection of EBV.

### Abortive Lytic Infection in the Pre-latent Phase Transitioned to Latent Infection

RNA-seq analysis at discrete time points during EBV infection shows the abortive lytic infection in the pre-latent phase, consistent with other studies (Kalla et al., 2010; Jochum et al., 2012a). However, because snapshot analyses, such as RNA-seq, lack temporal resolution, it has remained unclear whether the cells showing abortive lytic infection during the pre-latent phase are able to shift into latently infected cells.

**FIGURE 6:**
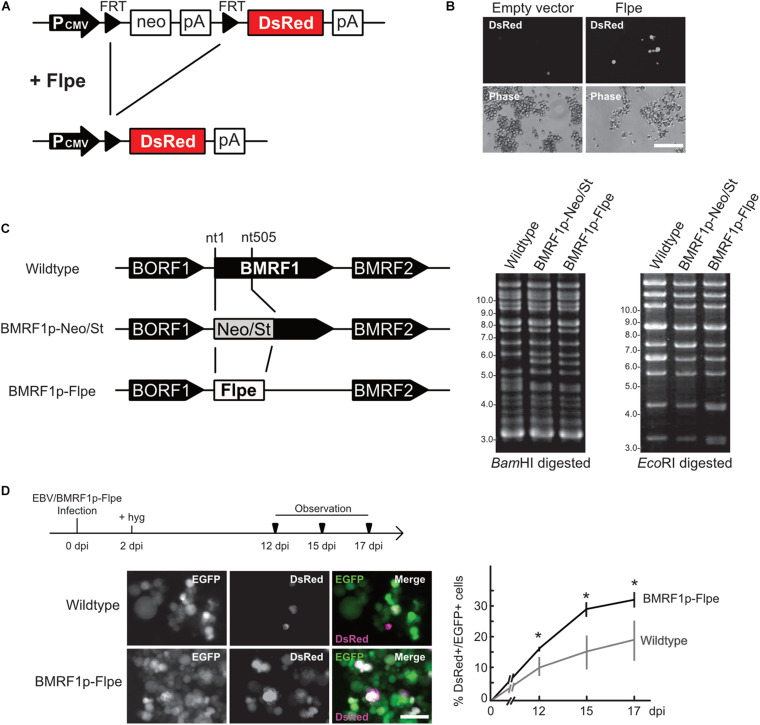
EBV establishes latency in B-cells via an abortive lytic infection in the pre-latent phase. **(A)** Schematic representation of the genetic elements in the Flpe-FRT system. Flpe recombinase can recombine FRT sites in the ubiquitously expressed reporter construct to remove the STOP (neomycin resistant gene and polyA signal; neo-pA) cassette. Upon removal of this STOP, the reporter DsRed is expressed in the cells and all their progeny. **(B)** DsRed was expressed in the presence of Flpe. Akata/FNF-DsRed reporter cells were transfected with a Flpe expression plasmid. Scale bar, 100 μm. **(C)** Schematic diagram of recombinant EBV (EBV/BMRF1p-Flpe) construction. The Neo/St cassette, containing neomycin resistance and streptomycin sensitivity genes, was inserted between nucleotides 1 and 505 of the BMRF1 gene to prepare an intermediate, and this was replaced with the Flpe sequence to construct the EBV/BMRF1p-Flpe, which expresses Flpe under the control of the BMRF1 promoter (*left*). Successful recombination was confirmed by the electrophoresis of the EBV-BAC after *Bam*HI and *Eco*RI digestion (*righ*t). **(D)** Continuous tracing of abortive lytic cells with recombinant EBV. Akata/FNF-DsRed cells were infected with EBV/BMRF1p-Flpe. Two days after infection, hygromycin was added to the medium to select infected cells. Infected cells were continuously observed (arrowheads) until 17 dpi. The number of DsRed-positive and EGFP-positive cells was counted at indicated time points. Results shown are the means ± SD of three independent experiments. Images were obtained at 17 dpi. Asterisk (*) indicates *p* < 0.05; dpi, days post-infection. Scale bar, 50 μm.

We, thus, applied fate mapping techniques using the Flippase (Flpe) recombinase-flippase recognition target (FRT) system (Kretzschmar and Watt, 2012) to trace the fate of cells exhibiting an abortive lytic infection in the pre-latent phase of EBV infection. Reporter cells were generated and isolated form Akata(−) cells, which transduced a red fluorescent protein (*DsRed*) reporter flanked by a neomycin-resistant gene containing a STOP codon (*FRT-STOP-FRT* sequence) (Figure 6A). In cells expressing both Flpe and the reporter, Flpe specifically activated the reporter by excising the STOP sequence. Indeed, we confirm the DsRed expression in the reporter cells in the presence of Flpe (Figure 6B). Notably, however, a few reporter cells expressed DsRed without Flpe expression. Furthermore, we generated the recombinant EBV(BMRF1p-Flpe), which expresses Flpe under the control of the BMRF1 promoter (Figure 6C). The *BMRF1* gene is categorized as an early lytic gene and encodes the DNA polymerase processivity factor, which associates with the polymerase catalytic subunit to enhance the polymerase processivity and exonuclease activity (Murayama et al., 2009). The BMRF1 protein is also known as early antigen diffused (EA-D) and is used as a clinical marker for EBV infection (Luka et al., 1986; Holley-Guthrie et al., 1990). Because the BMRF1 protein is abundantly expressed during lytic replication (Sugimoto et al., 2013), we chose the BMRF1 promoter for monitoring the EBV lytic gene expression. Infectious virus particles of EBV(BMRF1p-Flpe) were prepared by transient trans-complementation of BMRF1.

Using Akata/FNF-DsRed reporter cells and recombinant EBV, fate mapping of infected cells was performed. The reporter cells were infected with EBV(BMRF1p-Flpe) and were continuously observed during the pre-latent phase. In this system, EBV-infected cells were labeled by EGFP because recombinant EBV possesses eukaryotic promoter-driven EGFP. At 17 dpi, approximately 30% of infected cells, in which EBV had established latency, expressed DsRed (Figure 6D). This suggests a history of lytic gene expression in these cells during the pre-latent phase of EBV infection. Therefore, we found that the abortive lytic infection of EBV transitioned to latent infection during *de novo* infection of B-cells with EBV.

### BZLF1 Is Not Required for Abortive Lytic Infection in the Pre-latent Phase of EBV Infection

**FIGURE 7:**
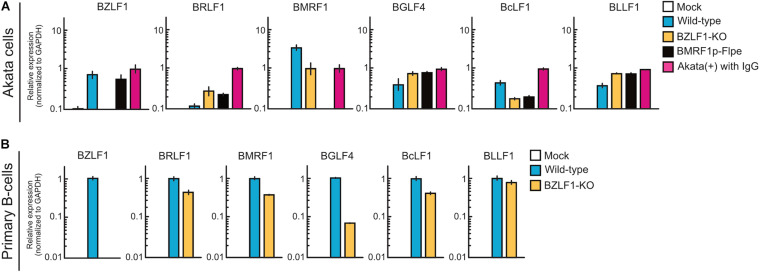
Abortive lytic gene expression of BZLF1-KO recombinant EBV in the pre-latent phase. **(A)** Akata(–) cells were infected with wild-type, BZLF1-KO, or BMRF1-KO (BMRF1p-Flpe) EBV. After 2 days, total RNA was extracted and analyzed by qRT-PCR. Results are presented as means ± SD of three independent experiments and are shown relative to gene expression in the lytic-induced Akata(+) cells after treatment with IgG. **(B)** Purified primary B-cells were infected with wild type or BZLF1-KO EBV and harvested at 3 dpi. EBV gene expression was assessed by qRT-PCR and normalized to GAPDH expression. Results are presented as means ± SD of three independent experiments.

The BZLF1 protein, a b-Zip transcriptional factor that binds to the promoters of early lytic genes (Farrell et al., 1989; Urier et al., 1989), triggers the switch from latent to productive lytic infection (Rooney et al., 1989). Upon EBV productive lytic infection, viral lytic genes are expressed in a strictly regulated temporal cascade involving the immediate-early, early, leaky late, and late phases. Except for BCRF1, BDLF2, and BDLF3, leaky late and late promoters are transactivated by the viral pre-initiation complex (Djavadian et al., 2018). Thus, to evaluate the difference in mechanisms underlying viral gene expression between pre-latent infection and productive lytic infection, Akata(−) cells were infected with wild-type, BZLF1-KO, or BMRF1p-Flpe (functional BMRF1-KO) EBV. At 2 dpi, the expression of viral lytic genes was detected in the cells infected with BZLF1-KO EBV, similar to wild-type EBV (Figure 7A), suggesting that BZLF1 was not essential for lytic gene expression in the pre-latent phase of EBV infection. Furthermore, BMRF1p-Flpe EBV infection revealed that these viral lytic genes, including late genes, such as BcLF1 and BLLF1, were expressed without viral replication during pre-latent infection (Figure 7A). We also confirm that BZLF1 was dispensable for abortive lytic gene expression in the pre-latent phase of EBV infection using primary B-cells infected with BZLF1-KO EBV (Figure 7B). It should be noted that another immediate-early gene, *BRLF1*, was not transcribed abundantly in the pre-latent phase (Figures 7A,B). These findings suggest that the regulation of viral gene expression in the pre-latent phase of EBV infection is different from that in the productive lytic phase.

## Discussion

EBV infects resting B-cells and transforms them into lymphoblastoid cell lines (LCLs) *in vitro*. LCLs share many common features with post-transplant lymphoproliferative disease and AIDS lymphomas (Shannon-Lowe et al., 2017). Thus, LCLs have been extensively used to study the mechanisms by which EBV causes cancers (Zhou et al., 2015; Jiang et al., 2017; Ma et al., 2017; Wang et al., 2019). However, because dynamic changes in viral infection overlap with the process involved in EBV-caused transformation during the infection of resting B-cells, transcriptomic changes between these two processes are indistinguishable. Here, we used EBV-negative cell lines derived from EBV-positive Burkitt lymphoma to evaluate the effect of EBV infection on host transcription in a time-course study. Using the Akata(−) cell line in this study enabled us to manipulate its genome for transducing our reporter system as discussed below. Similar to previous studies using primary B-cells (Mrozek-Gorska et al., 2019; Wang et al., 2019), our RNA-seq analysis on Akata(−) cells with EBV infection showed transient cell growth arrest for 2 dpi and re-proliferation thereafter (Figure 1C). These findings suggest that post-infectious growth arrest is associated with EBV infection but not with EBV-mediated transformation. Notably, this transient growth arrest after EBV infection is additionally reflected in our mathematical model of EBV infection (see section “Mathematical Modeling” in “Materials and Methods”).

Viral lytic genes were expressed transiently in newly infected cells during the pre-latent phase of EBV infection (Figures 2, 3). Because EBV does not initiate the *de novo* synthesis of progeny virus upon primary infection, the expression of these viral genes indicates an abortive lytic infection. However, comprehensive analyses of progeny production during the pre-latent phase had not been elucidated to date. In this study, our *in silico* simulation of EBV infection supports a lack of progeny virus production. Under the assumption that even a small amount of progeny virus is produced, the numbers of infected cells derived from our model were unable to trace the experimental data (Figure 4), providing theoretical evidence in support of our experimental observations as well as previous studies (Kalla et al., 2010, 2012).

RNA-seq data represents a snapshot at a single time point as a population average and lacks temporal resolution. Because the behavior of infected cells is highly dynamic and heterogeneous, these data miss the exact timing and order of events underlying infection. Continuous observation with cell tracking overcomes this obstacle. However, in this study, the low sensitivity of the reporter is one of the limitations (Figure 6B). When Flpe is expressed but remains below a certain threshold, DsRed expression cannot be induced. Thus, our system demonstrates EBV-infected cells in which transient lytic gene expression occurred qualitatively, not quantitatively. Despite these limitations, our fate mapping system with recombinant EBV successfully revealed that some cells display a record of lytic gene expression among the cells in which EBV established latency (Figure 6D). This finding suggests that the transient expression of lytic genes contributes to the establishment of latent infection in infected cells. In agreement with this, it has been reported that viral proteins BNLF2a and vIL-10/BCRF1 are expressed transiently upon the pre-latent phase of infection, and they prevent immune recognition and elimination of cells newly infected with EBV (Jochum et al., 2012a).

Our findings using recombinant EBV demonstrate that abortive lytic infection during the pre-latent phase is not required for BZLF1 expression in contrast to productive lytic gene expression (Figure 7). In cells latently infected with EBV, the viral genome is highly methylated; therefore, viral lytic genes are tightly suppressed (Fernandez et al., 2009). BZLF1 protein preferentially binds to CpG-methylated DNA motifs and enhances transcription more efficiently (Bhende et al., 2004), whereas the incoming EBV genome is barely methylated. These findings suggest that viral lytic genes might be expressed as leaky during the pre-latent phase in a BZLF1 protein-independent manner. DAVID analysis of our RNA-seq data also shows that promoters of upregulated genes in the pre-latent phase contain NF-κB binding sites (Table 3). The NF-κB pathway is activated during infection of B-cells with EBV (Jha et al., 2016) and considered to be a critical pathway in EBV-associated lymphomagenesis (Vento-Tormo et al., 2014; Battle-Lopez et al., 2016). In this sense, it may be purposeful that the NF-κB pathway is involved in lytic gene expression in the pre-latent phase of EBV infection. Further studies are required to understand the regulation of viral gene expression in the pre-latent phase.

**TABLE 3:** University of California Santa Tissue Factor Binding Site analysis of top 300 upregulated genes at 2 dpi.

Term	Count	*p*-value	FDR^*a*^
NFKAPPAB	126	7.26 × 10^–^^8^	1.28 × 10^–^^5^
NFKB	150	1.17 × 10^–^^4^	1.03 × 10^–^^2^
CETS1P54	76	7.76 × 10^–^^3^	0.351
BACH2	122	7.97 × 10^–^^3^	0.351

*^*a*^FDR, false discovery rate.*

In summary, combining the techniques of population-based averaged snapshot analysis and a continuous tracking system with fluorescent protein expression, we demonstrate herein that EBV establishes latency in B-cells via an abortive lytic infection in the pre-latent phase, implying the reversibility of the abortive lytic state during EBV infection. Our findings shed light on novel roles of EBV lytic genes in the initial, pre-latent phase of B-cell infection.

## Materials and Methods

### Cells

Akata(−) and Akata(+) cells (Shimizu et al., 1994) were cultured in RPMI1640 medium containing 10% fetal bovine serum (FBS). The productive lytic replication of Akata(+) cells was induced by treatment with anti-human IgG (Masud et al., 2017). AGS/EBV-EGFP cells were kindly provided by Dr. Hironori Yoshiyama (Katsumura et al., 2009) and were grown in F-12 HAM’s medium supplemented with 10% FBS and 400 μg/mL G418. HEK293 (a kind gift from Dr. Henri-Jacques Delecluse), HEK293T (ATCC CRL-3216), and HEK293/EBV-Bac cells were maintained in DMEM supplemented with 10% FBS. Primary B-cells (hPB CD19+ B-cells, negatively selected; 4W-601) were purchased from Lonza (Walkersville, MD, United States).

All cells were maintained at 37°C in an atmosphere of 5% CO_2_.

### Plasmids and Lentiviral Vector

To construct the lentiviral reporter plasmid (CSII-CMV-FNF-DsRed), the FNF-DsRed fragment was generated by PCR from pCAFNF-DsRed, a kind gift from Dr. Connie Cepko (Addgene plasmid #13,771), and was inserted into the *Bam*HI-*Xba*I site of the CSII-CMV-MCS-IRES2-Venus plasmid (generously gifted by Dr. Hiroyuki Miyoshi). The inserted DNA sequence was confirmed by direct DNA sequencing.

The lentiviral vector was prepared by recovering the culture supernatant of 293T cells transfected with CSII-CMV-FNF-DsRed together with expression plasmids for HIV-1 Gag-Pol and Rev (pCMVR8.74, Addgene plasmid #22,036; kindly provided by Dr. Didier Trono) and for VSV-G (generously provided by Dr. Yasuo Ariumi).

The Flpe expression plasmid (pCSFLPe) was the kind gift of Dr. Gerhart Ryffel (Addgene plasmid #31,130). pcDNA-BZLF1, pcDNA-gB, and pcDNA-BMRF1 were previously described (Sato et al., 2009; Nakayama et al., 2010; Konishi et al., 2018).

### Recombinant EBV-Bac

The original EBV-BAC DNA (B95-8 strain) was kindly provided by Dr. Wolfgang Hammerschmidt (Delecluse et al., 1998). To construct the BMRF1 promoter-driven Flpe expression EBV-Bac [EBV(BMRF1p-Flpe)], homologous recombination was undertaken in *E. coli* as described previously (Murata et al., 2009) with the oligonucleotide primers listed in Table 4. After construction of recombinant EBV-BAC strains, DNA was digested with *Bam*HI or *Eco*RI and resolved by agarose gel electrophoresis. BZLF1-KO EBV [EBV(dBZLF1)] was described previously (Sato et al., 2009).

**TABLE 4:** Oligonucleotide primers used for generation of recombinant EBV.

Primer name	Sequence (5′–3′)
BMRF1-Neo/st forward	GCATAAATTCTCCTGCCTGCCTCTGCTCTGGTACGTTGGCTTCTGCTGCTGCTTGTGATCGGCCTGGTGATGATGGCGGGATC
BMRF1-Neo/st reverse	CTTAACGCCGCCTGAGCCTTGCTGGCGTGCCCACTTCTGCAACGAGGAAGCCGTCTTGGGTCAGAAGAACTCGTCAAGAAGG
Forward transfer for Flpe	GCATAAATTCTCCTGCCTGCCTCTGCTCTGGTACGTTGGCTTCTGCTGCTGCTTGTGATCATGCCACAATTTGATATATT
Reverse transfer for Flpe	CTTAACGCCGCCTGAGCCTTGCTGGCGTGCCCACTTCTGCAACGAGGAAGCCGTCTTGGGCTATAGTTCTAGAATGCGTCTA

HEK293 cells were transfected with EBV-BAC DNA using Lipofectamine 2000 reagent (Thermo Fisher Scientific, Waltham, MA, United States) followed by hygromycin selection, and EGFP-positive cell colonies were selected for preparation of cell clones.

### EBV Preparation

EGFP-EBV was obtained from the 8-days-old, cell-free supernatant of AGS/EGFP-EBV cells. The cell-free supernatant was passed through 0.45 μm filters and then used as a virus stock.

For the preparation of recombinant EBV, HEK293/EBV (BMRF1p-Flpe), HEK293/EBV (dBZLF1), and HEK293/EBV (Wild-type) (Sato et al., 2019) cells were transfected with a BZLF1 expression plasmid together with the gB expression plasmid using the Neon Transfection System (Thermo Fisher Scientific) to induce lytic replication. Cells and culture supernatants were collected, freeze-thawed, and centrifuged. The supernatant from the centrifugation was filtered and used as a virus stock.

### RNA-Seq

Akata(−) cells were pelleted and resuspended in medium containing virus supernatant. Cells were incubated at room temperature for 2 h with agitation. The cells were spun again and resuspended in fresh medium. EBV-infected Akata cells that express EGFP (EGFP-positivity was ∼20% in the population at 2 dpi) were sorted by a FACS Aria II Cell Sorter (BD Biosciences, San Jose, CA, United States) at 2 dpi and then cultured. The cells were harvested for RNA preparation at later time points.

Total RNA was extracted using a Nucleospin RNA XS kit (Takara Bio, Kusatsu, Japan). Evaluation of RNA, RNA-seq library preparation, illumina sequencing, and data preprocessing were performed as described previously (Okuno et al., 2019).

Gene expression levels were normalized as counts per million (CPM) followed by log_2_-transformation with a pseudo-count of 1. The 5,000 most diversely expressed viral and host genes were used for downstream analyses. After standardization, Euclid distances among genes were calculated according to their expression patterns. Clustering analysis of the genes was performed using the Ward method based on the Euclid distances. All analyses were performed on R (version 3.5.2).

GO enrichment analysis was performed according to an overlap-based method with one-sided Fisher’s exact test. The family-wise error rate (i.e., adjusted *p*-value) was calculated using the Holm method. As a source of gene sets, we used “GO biological process” and “GO cellular component” obtained from the GO consortium (GO validation date: 08/30/2017)^1^.

Other functional annotation analyses were performed using DAVID Bioinformatics Resources^2^ (Huang et al., 2009).

### Data Collection for Mathematical Modeling

Growth curves were measured as described previously (Sato et al., 2017). Briefly, Akata(−) cells were seeded at a density of 1 × 10^5^ cells/mL. Every 48 h, the number of viable cells was counted. For EBV infection, 2 × 10^5^ cells of Akata(−) cells were pelleted and resuspended in 3 mL of medium containing EBV-EGFP [1 × 10^5^ green Akata units (GAU)]. The infected cells were seeded into a low-binding 35 mm dish (PrimeSurface dish MS-9035X; Sumitomo Bakelite, Tokyo, Japan) and maintained in a 37°C incubator at 5% CO_2_. The number of virus particles in the culture supernatant and the number of infected cells were routinely measured as follows: a portion (400 μL) of the infected cell culture was routinely harvested, and the amount of released infectious virions in the culture supernatant was quantified as described previously (Sato et al., 2019). The cell number was counted as described previously (Sato et al., 2017). The percentage of infected cells was quantified by FACS (Sato et al., 2019).

For virus decay analysis, EBV-EGFP (1 × 10^5^ GAU) was suspended in 3 mL of medium and incubated (37°C/5% CO_2_). Samples (100 μL) were routinely harvested, and the virus titer was quantified as described previously (Sato et al., 2019).

### EBV Infection to Primary B-Cells

A total 5 × 10^6^ cells of primary B-cells were pelleted and resuspended in medium containing virus supernatant at a multiplicity of infection of 3 GAU. Cells were incubated at room temperature for 2 h with agitation. The cells were spun again, resuspended in fresh medium, and then cultured. The cells were harvested for RNA preparation at later time points.

### Growth Kinetics of Akata Cells and Viral Decay Kinetics

We independently estimated the growth kinetics of Akata cells by the following mathematical model (Eq. 5) from separate experiments (see growth curve section in “Data Collection for Mathematical Modeling”):

(5) $$\frac{d\,T\,\left(t\right)}{d\,t}=g\,T\,\left(t\right)\,\left(1-\frac{T\,\left(t\right)}{K}\right)$$

Here, the variable *T*(*t*) is the number of Akata cells, and the parameters *g* and *K* are the growth rate and the carrying capacity of the cell culture, respectively. We also estimated the viral decay kinetics by the following Eq. (6) from separate experiments (see virus decay section in “Data Collection for Mathematical Modeling”):

(6) $$\frac{d\,V\,\left(t\right)}{d\,t}=-c\,V\,\left(t\right)$$

Here, *V*(*t*) is the number of virions, and *c* represents the viral decay rate. These parameters, *g*, *K*, and *c*, are fixed hereafter.

### Fate Mapping of EBV-Infected Cells

For generating reporter cells, Akata(−) cells were inoculated with the lentiviral vector harboring the CMV promoter-driven FNF-DsRed cassette and followed by G418 selection (750 μg/mL).

Akata/FNF-DsRed cells were incubated in medium containing EBV(BMRF1p-Flpe) at room temperature for 2 h with agitation. The cells were spun down, resuspended in fresh medium, and then cultured. Expressions of fluorescent proteins, EGFP, and DsRed were observed at discrete time points. Images were acquired using a Zeiss Axio Observer microscope (Carl Zeiss, Oberkochen, Germany).

### Quantitative Reverse-Transcription PCR (qRT-PCR)

Total RNA was prepared using a Nucleospin RNA XS kit (Takara Bio) or RNeasy mini kit (Qiagen, Gaithersburg, MD, United States) and, subsequently, reverse transcribed to cDNA using the PrimeScript II Reverse transcriptase kit (Takara Bio). Viral mRNA levels were analyzed by qPCR using the 7500 Fast DX Real-Time PCR system (Applied Biosystems, Foster City, CA, United States) as previously described (Watanabe et al., 2015; Sato et al., 2017). The original primer sequences used in this study are listed in Table 5.

**TABLE 5:** Oligonucleotide primers used for qRT-PCR.

Primer name	Sequence (5′–3′)
EBNA1 forward	GATTCTGCAGCCCAGAGAGT
EBNA1 reverse	TCTCTCCTAGGCCATTTCCA
EBNA-LP forward	CCCCTCTCTCTGTCCTTCAG
EBNA-LP reverse	GGCTCCCCTCAGACATTCTT
LMP1 forward	CTGATGATCACCCTCCTGCT
LMP1 reverse	CTAAGACAAGTAAGCACCCGAAG
BZLF1 forward	GAAGCACCTCAACCTGGAGA
BZLF1 reverse	TCTGGCTGTTGTGGTTTCC
BRLF1 forward	TCATTAAGTTCGGGGGTCAG
BRLF1 reverse	GGACCCTGATGAAGAAACCA
BGLF4 forward	TGACGGAGCTGTATCACGAG
BGLF4 reverse	CCAGGGGCTCAATACTACCA
BMRF1 forward	ATTTTACAAGCGGCCACAAG
BMRF1 reverse	CCAATCATCTGCTCGTTCCT
BHRF1 forward	AAATGGTACCCTGCATCCTG
BHRF1 reverse	CCACATGTTCGGTGTGTGTT
BcLF1 forward	AGGTTGGGAGGAAAACGTAG
BcLF1 reverse	TTAACGGAGACCACGACCAC
BLLF1 forward	CCCTCACTACTGCCGTTATA
BLLF1 reverse	GCCTGGAATCTGTAGATGTC
GAPDH forward	CCTCCAAGGAGTAAGACCCC
GAPDH reverse	TGTGAGGAGGGGAGATTCAG

### Statistical Analysis

Results are shown as the means ± SD of three independent experiments. Statistical analyses were performed using Microsoft Excel and R (version 3.5.2). Unpaired Student’s *t*-test was used to determine significance between two groups. *p* < 0.05 were considered significant.

## Data Availability Statement

The datasets presented in this study can be found in online repositories. The names of the repository/repositories and accession number(s) can be found below: https://www.ddbj.nig.ac.jp/, DRA009706.

## Author Contributions

YS, TM, and HK led the entire project. TI, YS, JI, MT, YO, MY, HM, and TW performed the research. YS, JI, MT, YO, SI, and KS analyzed the data. TI, YS, JI, MT, SI, KS, and HK wrote the manuscript. All authors reviewed the manuscript for its content.

## Conflict of Interest

The authors declare that the research was conducted in the absence of any commercial or financial relationships that could be construed as a potential conflict of interest.

## Footnotes

1 http://geneontology.org/

2 https://david.ncifcrf.gov/
