# Corrigendum: Direct evidence of abortive lytic infection-mediated establishment of Epstein-Barr virus latency during B-cell infection

**DOI:** 10.3389/fmicb.2024.1426311

**Published:** 2024-05-23

**Authors:** Tomoki Inagaki, Yoshitaka Sato, Jumpei Ito, Mitsuaki Takaki, Yusuke Okuno, Masahiro Yaguchi, H. M. Abdullah Al Masud, Takahiro Watanabe, Kei Sato, Shingo Iwami, Takayuki Murata, Hiroshi Kimura

**Affiliations:** ^1^Department of Virology, Nagoya University Graduate School of Medicine, Nagoya, Japan; ^2^Precursory Research for Embryonic Science and Technology (PRESTO), Japan Science and Technology Agency, Kawaguchi, Japan; ^3^Division of Systems Virology, Department of Infectious Disease Control, International Research Center for Infectious Diseases, Institute of Medical Science, The University of Tokyo, Tokyo, Japan; ^4^Mathematical Biology Laboratory, Department of Biology, Faculty of Sciences, Kyushu University, Fukuoka, Japan; ^5^Medical Genomics Center, Nagoya University Hospital, Nagoya, Japan; ^6^Department of Microbiology, Faculty of Biological Sciences, University of Chittagong, Chattogram, Bangladesh; ^7^Core Research for Evolutional Science and Technology (CREST), Japan Science and Technology Agency, Kawaguchi, Japan; ^8^MIRAI, Japan Science and Technology Agency, Kawaguchi, Japan; ^9^Department of Virology and Parasitology, Fujita Health University School of Medicine, Toyoake, Japan

**Keywords:** EBV, pre-latent phase, abortive lytic infection, fate mapping, neo virology

In the published article, there was an error in Table 2 as published. We have carelessly swapped the rows and columns of Table 2. The corrected Table 2 and its caption appear below.

**Table 2:** Estimated parameters by fitting the mathematical model to experimental data.

**Symbol**	**Unit**	**Value**		
		***p* = 0**	***p* = 0.1**	***p* = 1,000**
*g*	Day^−1^	7.33 × 10^−1^		
*K*	Cell	6.51 × 10^6^		
*c*	Day^−1^	2.44 × 10^−1^		
β	(Day × cell)^−1^	2.46 × 10^−6^	6.36 × 10^−7^	8.08 × 10^−11^
*T*_0_	Cell	8.53 × 10^4^	6.76 × 10^4^	6.17 × 10^4^
*I*_*ar*, 0_	Cell	1.80 × 10^4^	3.88 × 10^4^	4.62 × 10^4^

The authors apologize for this error and state that this does not change the scientific conclusions of the article in any way. The original article has been updated.

